# Systemic Strategies to Prevent Nonbeneficial Treatments Near the End of Life

**DOI:** 10.1001/jamanetworkopen.2025.19771

**Published:** 2025-07-10

**Authors:** Sofia Weiss Goitiandia, Amy Z. Sun, Amy Rosenwohl-Mack, Catthi Ly, Katherine E. Sleeman, Daniel Dohan, Elizabeth Dzeng

**Affiliations:** 1 Division of Hospital Medicine, University of California, San Francisco; 2 Department of Geriatrics and Extended Care, Division of Palliative and Hospice Care, Tibor Rubin VA Medical Center, Long Beach, California; 3 Division of Hospice and Palliative Medicine, University of California, Irvine, Orange; 4 Cicely Saunders Institute, King’s College London, London, United Kingdom; 5 Philip R. Lee Institute for Health Policy Studies, University of California, San Francisco

## Abstract

**Importance:**

There exists a default toward high-intensity treatments near the end of life in the United States, including for people living with advanced dementia (PLWD). Clinical momentum, a cascade of increasingly intensive treatments facilitated by systemic factors, contributes to this default. The intensity of treatments provided to PLWD near the end of life is lower in Great Britain. Using Great Britain as a counterexample to the United States, this study examines factors that may contribute to lower-intensity treatment patterns.

**Objective:**

To understand factors within the British health care system that shape treatment intensity for PLWD.

**Design, Setting, and Participants:**

This qualitative study used semistructured, in-depth interviews with clinicians at 1 National Health Service (NHS) trust in South London and with caregivers of PLWD in England and Wales. Interviews were conducted between February 2021 and February 2023. Perspectives on factors influencing treatment escalation decisions in PLWD were elicited. Data were analyzed using thematic analysis.

**Main Outcomes and Measures:**

The primary outcome was the individual-, institutional-, and system-level factors that affect treatment escalation decisions among PLWD in Great Britain.

**Results:**

A total of 13 clinicians (11 [84.6%] women; 3 [23.1%] Asian or Asian British, 1 [7.7%] Black, Black British, Caribbean, or African, and 9 [69.2%] White; median [range] years in practice, 26 [8-35]) and 14 caregivers (8 [57.1%] women; 3 [21.4%] Asian or Asian British, 2 [14.3%] Black, Black British, Caribbean, or African, and 8 [57.1%] White; median [IQR] age among 13 who provided data, 32 [28-45] years) participated. Caregiver respondents discussed individual-level factors preventing escalation to high-intensity treatments (transparent communication and knowledge of dementia trajectory). Clinician and caregiver respondents described institutional-level factors (eg, protocols, resources, and practices) and system-level factors (eg, national policies, laws, and cultural norms) shaping treatment escalation decision-making.

**Conclusions and Relevance:**

In this qualitative study of clinicians and caregivers in Great Britain, respondents reported individual-, institutional-, and system-level factors that they perceived to both independently and collectively prevent potentially nonbeneficial treatment escalation, including to life-sustaining treatments, and facilitate deescalation in PLWD. These factors created structured opportunities for clinicians and caregivers to deliberate on decisions to escalate treatment intensity. The convergence of these factors generated a clinical practice pattern minimizing escalation toward high-intensity treatments in PLWD, referred to here as *clinical deceleration*, in contrast to the default clinical momentum observed in the United States.

## Key Points

**Question:**

What individual-, institutional-, and system-level factors do clinicians and caregivers perceive as shaping treatment intensity for people living with advanced dementia (PLWD) in Great Britain?

**Findings:**

In this qualitative study with 27 participants, respondents described individual-, institutional-, and system-level factors influencing treatment escalation decision-making. Mutually reinforcing factors across levels resulted in a clinical practice pattern favoring limited treatment escalation.

**Meaning:**

In this study, treatment escalation decisions for PLWD in Great Britain were shaped by individual-, institutional-, and system-level factors that oriented practice toward lower-intensity treatments.

## Teaser

This qualitative study evaluates what factors clinicians and caregivers of people living with dementia in Great Britain perceive to shape treatment intensity toward the end of life.

## Introduction

The US and British health care systems differ in their use of high-intensity, life-sustaining treatments (LSTs) for older adults.^1,2^ Great Britain has lower rates of intensive care unit (ICU) admission and mechanical ventilation than the United States.^1,2,3,4^ High-intensity LSTs are a concern among people living with advanced dementia (PLWD), as evidence suggests they offer limited survival benefits. For instance, increased utilization of mechanical ventilation did not improve 1-year mortality rates in PLWD.^3^ High-intensity LSTs may also conflict with the preferences of some PLWD and their surrogates,^5^ resulting in lower-quality, goal-discordant care.^6^ Given that PLWD often lack decisional capacity near the end of life, it is particularly important to understand how clinicians and caregivers make decisions about unwanted treatment escalation on their behalf.^7^

Factors at various health care system levels have been shown to shape intensity of end-of-life care. These include factors at the individual (eg, patient preferences, clinician behavior), institutional (eg, hospital policies), and system (eg, legal and cultural contexts, health care system financing and structure) levels.^1,2^ Our prior US-based work demonstrated how mutually reinforcing relationships between institutional structures (eg, policies) and cultures (ie, shared values and practices influencing operations) promoted norms leading to higher-intensity treatments.^8,9,10^ *Clinical momentum* describes treatments added in a cascading manner, bypassing opportunities to deliberate upon their value.^11^

Existing literature has emphasized individual-level factors over social-level factors, concentrating, for example, on physicians’ influence over admitting older adults to emergency departments^12^ or enrolling PLWD in hospice.^13^ While physician characteristics are important, serious illness care for PLWD often requires multidisciplinary collaboration across the hospital.^14^ Research on institutional- and system-level contexts is thus required to elucidate factors shaping treatment escalation fully. Identifying social-level influences could reveal levers to prevent clinical momentum, critical given the limited success of individual-level interventions.^15,16^

We examined how factors across different levels of the health care system might shape treatment escalation decision-making in Great Britain. We hypothesized that Great Britain could serve as a counterexample to the clinical momentum pattern documented in the United States.

## Methods

### Study Design, Setting, and Participants

For this qualitative study, we conducted semistructured, in-depth interviews to explore clinicians’ and caregivers’ perspectives on factors influencing treatment escalation for PLWD. Interviews were conducted with clinicians at 1 National Health Service (NHS) trust in South London and with caregivers in England and Wales. South London is a major metropolitan area with a well-resourced public health care system and a robust network of palliative care services. This NHS trust was selected for its status as an academic medical center, broadly representative of those in London offering tertiary care.^17^ It is situated in an ethnically and socioeconomically diverse area.^17^

We used purposive sampling to capture clinician perspectives from various specialties and settings.^18,19^ Clinicians were recruited through clinical listservs, professional networks, and direct email solicitation, using snowball sampling for interviewees to identify further clinician roles of interest. Caregivers were recruited across Great Britain; eligibility criteria included caring for a PLWD or being bereaved within 2 years. A recruitment e-mail, developed with community input through a national dementia organization, was disseminated through this organization and a London palliative care research center. The researchers and center also disseminated the notice through social media.

The NHS Health Research Authority and Research Ethics Council granted ethical approval. Informed consent was obtained from all participants, including to publish deidentified quotations. All participants self-reported their sociodemographic characteristics using UK government-endorsed racial and ethnic categorizations.^20^ Race and ethnicity data were collected as we aimed to be cognizant of including diverse participants and comprehensively recording demographic information. We report our study using the Standards for Reporting Qualitative Research (SRQR) guideline.^21^

### Data Collection

E.D. conducted interviews in person or by videoconferencing between February 2021 and February 2023. The research team developed different interview guides to capture perspectives unique to clinicians and caregivers, drawing on the literature and our prior research. The guides elicited insights related to individual, institutional, and health care system levels, emphasizing the latter 2, aligning with our study’s focus. We constructed a comprehensive view of the system’s role in directing escalation decisions by synthesizing data from individuals at different vantage points.^22^ Interviews were audiotaped, transcribed, and anonymized. Data collection and analysis occurred concurrently and iteratively, refining the guides based on earlier interviews. Interviews occurred until theoretical saturation, when further interviews generated no new insights.^23,24^

### Data Analysis

We used thematic analysis to generate themes from our data.^25,26^ Three researchers (E.D., A.R.-M., and A.Z.S.) independently and group-coded a subset of clinician and caregiver interviews using thematic coding^25,27,28^ and, after initial coding, deductively and inductively developed preliminary codebooks for each cohort.^29,30^ Deductive coding was guided by the institutional and system-level factors developed in our prior research (eg, policies, practices, protocols, resources, and culture).^8,9,10^ Twenty percent of interviews were double- or group-coded by 2 or more researchers, refining the codebooks. Three team members (A.Z.S., A.R.-M., and C.L.) coded the remaining interviews, with disagreements addressed through discussion of code definitions until consensus. We used ATLAS.ti version 23.4.0 (ATLAS.ti Scientific Software Development) for data management.

Following coding, A.Z.S., S.W.G., C.L., and E.D. developed overarching themes. We isolated codes from both codebooks addressing treatment escalation and decision-making. We compared transcript sections with these codes applied from the 2 cohorts to identify thematic convergences and divergences. Our approach acknowledged that multiple perspectives are intrinsic and that researchers’ positionalities influence data interpretation. A strength of the analysis team (A.Z.S., E.D., S.W.G., C.L., A.R.-M.) was our diverse professional perspectives, comprising clinicians (2 physicians, A.Z.S. and E.D., and a nurse, A.R.-M.) and researchers (S.W.G. and C.L.), 3 of whom (E.D., S.W.G., and A.R.-M.) had experience in both British and US health care systems. All researchers reflected on how our backgrounds influenced our interpretations. To mitigate bias and increase rigor, we paid attention to counterfactual data, using these to refine themes. Findings were validated through triangulation and expert review by a US social scientist (D.D.) and a British palliative care physician-researcher (K.E.S.).

## Results

A total of 13 clinicians (11 [84.6%] women; 3 [23.1%] Asian or Asian British, 1 [7.7%] Black, Black British, Caribbean or African, 9 [69.2%] White individuals; median [range] years in practice, 26 [8-35]) and 14 caregivers (8 women [57.1%]; 3 [21.4%] Asian or Asian British, 2 [14.3%] Black, Black British, Caribbean or African, 1 [7.1%] mixed or multiple ethnic groups, 8 [57.1%] White individuals) were interviewed (Table 1). The median (IQR) age among the 13 caregivers who provided data was 32 (28-45) years.

**Table 1.** Clinician and Caregiver Respondent Sociodemographic Characteristics

Characteristic	No. (%)
Clinicians, total No.	13
Caregivers, total No.	14
Clinician self-identified gender	
Man	2 (15.4)
Woman	11 (84.6)
Caregiver self-identified gender	
Man	6 (42.9)
Woman	8 (57.1)
Clinician self-reported racial identity	
Asian or Asian British	3 (23.1)
Black, Black British, Caribbean or African	1 (7.7)
White	9 (69.2)
Caregiver self-reported racial identity	
Asian or Asian British	3 (21.4)
Black, Black British, Caribbean or African	2 (14.3)
Mixed race or multiple ethnic group	1 (7.1)
White	8 (57.1)
Type of clinician	
Physician	
Overall	5 (38.5)
Level of training	
Registrar (resident equivalent)	2 (15.4)
Consultant (attending equivalent)	3 (23.1)
Nurses	
Overall	7 (46.2)
Type of nurse	
Clinical nurse specialist	2 (15.4)
Nurse practitioner	2 (15.4)
Frailty practitioner	1 (7.7)
Nursing consultant	2 (15.4)
Other clinicians	
Matron	1 (7.7)
Clinical specialty	
General practitioner (primary care physician)	2 (15.4)
Acute medicine	1 (7.7)
Dementia	2 (15.4)
Mental health	1 (7.7)
Geriatrics or frailty care for older adults	4 (30.8)
Palliative care	6 (46.2)
Multiple specialties^a^	3 (23.1)
Caregivers’ relationship to PLWD	
Child of PLWD	10 (71.4)
Grandchild of PLWD	1 (7.1)
Spouse of PLWD	1 (7.1)
Other family member of PLWD	2 (14.3)
Caregivers’ region	
London	2 (14.3)
South East England (excluding London)	2 (14.3)
North East England	1 (7.1)
West Midlands	2 (14.3)
East Midlands	3 (21.4)
Wales	1 (7.1)
East England	1 (7.1)
North West England	1 (7.1)
South West England	1 (7.1)
Clinicians’ years of experience, median (range)	26 (8-35)

Abbreviation: PLWD, people living with advanced dementia.

^a^ Multispecialty clinicians are reported twice in the table, thereby leading to a sum percentage over 100%.

### Individual-Level Factors

Individual-level factors included attitudes, beliefs, and behaviors regarding treatment escalation. Only caregiver respondents spoke to the impact of individual-level factors, drawing from personal encounters with clinicians. In contrast, clinicians emphasized institutional and systemic considerations, likely due to their professional roles and proximity to these social-level factors.

#### Clinician Communication

Caregiver respondents felt escalation and deescalation were facilitated by clinicians’ transparent communication. Respondents described positive communication experiences, such as when a physician candidly discussed prognosis and recommended deescalation (quotation [Q] 1 in Table 2). This approach enabled consensus to deescalate (Q1). Conversely, caregiver respondents described how insufficient information about treatments and anticipated outcomes led to unwanted escalation and caregiver regret (Q2).

**Table 2.** Multilevel Factors That Were Perceived to Prevent Escalation or Facilitate Deescalation

Theme	Respondent	Quotation	Quotation No.
**Individual-level factors**			
Transparent communication by clinicians facilitated decisions to deescalate high-intensity treatments	Caregiver 7	“The consultant said, ‘We’re never going to achieve the goal we should to get her [mother] out [of hospital]. Where do you want her to go?’ The diagnosis was there, the prognosis was there, it was just somebody taking the time to act humanely and agree she wasn’t in the best place to die.”	1
	Caregiver 4	“The doctor said, ‘If we operate, she’ll be able to walk a lot better.’ We had no idea about her recovery time. She had no rehabilitation. If the doctor had told us what was involved, I could have convinced my grandparents that the plasters would have been the better choice.”	2
Understanding the dementia disease trajectory empowered caregivers to choose to deescalate treatments	Caregiver 1	“[The clinicians] found she had rectal cancer. They started talking about putting stents in her bowel. I thought, ‘She’s 91. She’s got advanced dementia.’ I said, ‘Can we talk about the elephant in the room? Palliative care.’ And you could see their shoulders kind of like, ‘Phew.’”	3
	Caregiver 7	“We [caregiver and clinician] put together her age, dementia, and [that] she’s not eating and drinking. I started to read and speak to aging-focused organizations. [The clinician’s] mindset was like mine: that she was never going to get fit enough to be discharged. I was expressing my real concern that I wanted her out of hospital. [The clinician] said, ‘We’ll discharge her.’”	4
**Institutional-level factors**			
Clinical scores and ICU activation pathways assisted in recognizing clinical decompensation and guided ICU admission decisions	Palliative care nursing consultant 1	“[The ward nurses give a] score depending on how outside of a normal range somebody is [when doing observations]. They… might escalate it to the [ICU outreach team]. They will look in detail at the patient and say, ‘This is not the sort of patient we’re going to take to critical care,’ and underline the decision.”	5
	Palliative care nurse 1	“When [a patient’s] observation scores are off baseline, the level of care needs to be established. The [ICU outreach team] will assess the patient and give their opinions to the team, and they discuss that with the family or the patient and decide whether they want to take them to a high-dependency unit or ICU care.”	6
Frailty practitioners served as a resource for supporting treatment escalation decisions in the A&E department	Frailty practitioner 1	“I’m conveying [to clinical teams] a comprehensive geriatric assessment. It’s everything I know… think, and maybe advice on ‘We don’t need to keep them in. These are services to get them home quicker.’ Sometimes there’s a knee-jerk reaction because [the patient’s] more confused, [clinicians think] they need to come in. No, we can do other things.”	7
	Geriatric nursing consultant 1	**“**With frailty practitioners seeing people at the front door, not only can you prevent unnecessary admissions, but… the people looking after the patient on the ward have the information to help them make decisions much sooner in the patient’s journey.”	8
	Frailty practitioner 1	“I’m digging up notes, calling families, carers, care homes. Someone can be in the ED for hours. They get moved to a ward, and nobody starts getting collateral until… 24 hours later. If I start getting it from the minute they come through the door... you could potentially reduce [admission] by 24 hours.”	9
Timely and coordinated dementia care prevented unnecessary hospitalizations	Palliative care physician 2	“The ‘@home’ team will do acute interventions in a person’s home for a limited time. If somebody comes into hospital, we might be able to get them out quite quickly, supported by ‘@home’ if they’re clear they don’t want to be in an acute bed.”	10
	Geriatric nursing consultant 1	“What’s improved in Enhanced Health in Care Homes has been having GPs… looking after all the care homes or having specialist care home GPs. They’re highly specialized and motivated to make sure residents have care plans.”	11
	Geriatric physician 1	“The idea of [Enhanced Health in Care Homes] is the multidisciplinary proactive management of older people in care homes… partly with a view to avoiding unplanned hospital admissions.”	12
**System-level factors**			
Policies empowered paramedics to discuss and implement decisions not to escalate treatments in the field	Palliative care physician 2	“The doctor might get a call [from London Ambulance]… saying, ‘I’m in this person’s house, and I see they’ve got a[n electronic urgent care plan] record. This is the situation. What do you think?’ They make efforts to… manage a crisis in the community as opposed to, as a default, bringing somebody to hospital.”	13
	Geriatric physician 1	“Ambulance crews have been pragmatic about saying, ‘Hospital is not going to be in this person’s best interest.’ I think because they’ve empowered the care plan. If you’ve got a clear, documented care plan, that gives… a lot of confidence to enact that.”	14
	Caregiver 5	“[The paramedic said,] ‘In end-of-life care, this is what we see… would you like your mum to go to hospital or stay where she is?’ And then the more he was talking, I thought, ‘No, that’s just prolonging the inevitable.’… I said, ‘I don’t want mum to go, I want her to stay in the care home,’… then I thought, ‘Here we go, I haven’t got power of attorney, oh my God.’ [Instead] he went, ’That’s fine. She certainly can [stay at the care home].’”	15
	Caregiver 3	“On one occasion, the ambulance people said, ‘Have you got a DNR in place? Because you wouldn’t want her to be resuscitated.’ They talked about how they’d have to break ribs and jump up and down. We all agreed, the family agreed, we wouldn’t want that.”	16
Shared electronic record systems conveying patients’ preferences aided goal-concordant care	Dementia nurse 1	“When you go into [the EPaCCS record], it’ll tell you whether they’ve got a DNACPR, it’ll tell you a ceiling of care. I can think of at least two instances where they’ve been kept at home [because of the record].”	17
	Geriatric physician 1	“If you go on [EPaCCS], there will be an alert to say [the advanced care plan is] there for anyone that has an interaction with that patient…. We are able to recognize that somebody is on a trajectory to dying,… [and we work] with care home staff to ensure the actions they take when somebody deteriorates fit with those care plans. In an ideal world, the ambulance wouldn’t even be called.”	18
Treatment escalation plans enabled the implementation of treatment limitations	Palliative care nursing consultant	“Treatment escalation plans are really helpful. When someone comes into hospital, if they already have complexities, lots of comorbidities, immediately senior medics in A&E are going to make a call on the level of support they should receive.”	19
	Palliative care physician 1	“The treatment escalation plan is… where you think about how appropriate it is for a person to escalate beyond ward-based care. We don’t say, ‘There is a limit on your care.’ We say, ‘We will never stop caring for you. But there is a limit to the treatments that will reverse this situation.’”	20
Legal and policy frameworks outlined clinician-led best interests decision-making	Palliative care physician 2	“Patients and families can’t demand treatments that doctors feel are not in their best interests. We have national guidance, and we have a law [the Mental Capacity Act] that stipulates that these are medical decisions. But best practice is that patients and families are involved in those decisions.”	21
	Geriatric physician 1	“Not providing an intervention is not a decision for a patient or a family to make. It’s a medical decision based on weighing the risks and benefits.… Somebody with known advanced dementia wouldn’t be intubated at the request of their families.”	22
	Palliative care physician 1	“Legally, the decision rests with the most senior clinician and the multidisciplinary team consensus. In this country… the law is on the doctor’s side. Patients and the families don’t often test that.”	23
	Geriatric physician 1	“The medico-legal background and ethical guidance from the General Medical Council is integral to the practice of all doctors in the UK because they are our registering body.… Doctors are not forced to give a treatment they think is not going to work. That underpins our ethical background.”	24
	Caregiver 1	“We respect doctors. We trust them. I think, initially, it has to be the doctor [who has decision-making priority] because they have to state the situation objectively. They have an acknowledged role in society, and they have the factual knowledge.”	25
	Caregiver 4	“I’d trust a doctor’s judgment. If a doctor said to me, ‘This is going to be the end of your mum’s life. There’s going to be no quality of life,’ then I wouldn’t want that. But I would still hope they would have that discussion with the family, so that they felt that mum was being heard.”	26
	Caregiver 8	“When I was not there, they would make decisions about… my father without trying to understand his care needs or speaking to me…. They were only concentrating on the medications and medical needs, not on his well-being needs.”	27
	Caregiver 10	“When you work in a place that is so multicultural; you have to be open to ways of thinking you may not agree with, or think are valid…. The hospital was used to… a certain type of family member. They expected me to put them on a pedestal and agree with everything they said.”	28
	Caregiver 10	“Sometimes [advocating] is more headache than it’s worth. Just having to listen to what [doctors] think should happen. And the stuff we think, because of our religion, we can’t advocate for.”	29
A tendency toward lower treatment intensity for PLWD	Geriatric physician 1	“Care home residents shouldn’t be coming into the hospital very much. I want to avoid people going into hospital, dying in an ambulance, dying in A&E, because that’s an undignified way to end your life.”	30
	Geriatric physician 1	“If [the patient] is at a level of dementia where they are not caring for themselves or bedbound and in a nursing home, very few of the ICU team would accept them.… Provide some oxygen outside of intensive care. But we probably stop there.”	31
	Palliative care registrar 2^a^	“It’s unusual for patients with dementia not to have other comorbidities that mean they are not ICU candidates. Hopefully, the other reason we don’t see them there is because there has been some planning about, ‘Is that the right place for them?’ It’s common for patients to have ward-based care as their ceiling.”	32
	Caregiver 3	“They wouldn’t put her on a ventilator because [mum] was too frail, which she wasn’t.… Two paramedics took her to the hospice. [The doctor] whispered to my brother, ‘She’ll be dead by tomorrow.’ But in the end, it was four days, and she developed pneumonia and died. My assumption is they decided it was time not to address the bronchiectasis, and… she should go.”	33
	Caregiver 4	“They didn’t do anything [in hospital]. No OT. We were going to have a meeting with the consultant to decide what our options were. We all got to the hospital, and the consultant left for the day. We were left in limbo…. When she moved to [town] [after discharge], the OT did an assessment and said mum will never walk again because she’d been left in bed for so long.”	34
Pragmatic concerns about resource allocation influenced approaches to treatment escalation	Geriatric practitioner 1	“There’s a huge push for avoiding hospital admissions from care homes, from a resource perspective. The NHS is looking for ways to improve efficiency and some… are around good anticipatory care. Not leaving everyone to reach crisis and then trying to deal with it but anticipating that these things will happen and working out ways to manage them.”	35
	Caregiver 14	“We used to have 16 palliative care beds, which closed when the hospital closed. The only palliative care we have is a mobile [and] a bit on the cancer side. It’s very difficult to get in because 30 beds are commissioned for 1 000 000 people.… The ward he ended up on for the last three weeks of his life provided palliative care, even though they weren’t experts at it.”	36

Abbreviations: A&E, accident and emergency; DNACPR, do not attempt cardiopulmonary resuscitation; DNR, do not resuscitate; ED, emergency department; EPaCCS, Electronic Palliative Care Coordinating Systems; GP, general practitioner; ICU, intensive care unit; NHS, National Health Service; OT, occupational therapy; PLWD, people living with advanced dementia.

^a^ A registrar is approximately the same as a resident in the UK medical system.

#### Knowledge of Dementia Trajectory

Knowledge of the dementia trajectory assisted caregiver respondents in advocating for deescalation. Based on their understanding of advanced dementia and multimorbidity, one caregiver steered discussions toward avoiding goal-discordant escalation (Q3 in Table 2). Another caregiver identified end-stage dementia signs and coordinated with clinicians to deescalate treatments (Q4).

### Institutional-Level Factors

Institutional-level factors encompassed policies, practices, protocols, resources, and hospital culture. Whether articulated in formal policies or kept implicit, participants believed that these elements influenced institutions’ decision-making practice patterns. Primarily clinician respondents described institutional factors.

#### Pre-ICU Pathways

Clinician respondents described how pre-ICU protocols and structures, like ICU outreach teams, supported holistic assessments and collaborative ICU admission decisions. While clinical scoring systems and ICU outreach teams were activated for patient decompensation, respondents emphasized that triggers did more than prompt automated responses. They created an opportunity for evaluation and discussion with families, where outreach teams could provide authoritative guidance on the utility of ICU-level treatments (Q5 and Q6 in Table 2). Ultimately, these pathways facilitated clinician-led decisions that prioritized patient goals within a best-interest decision-making framework (Q5 and Q6).

#### Frailty Practitioners

Clinician respondents identified frailty practitioners—nurses or physiotherapists specializing in working with frail adults—as an institutional resource for guiding hospital admission decisions. By collecting collateral information and assessing functional status in accident and emergency (A&E; equivalent to a US emergency department [ED]), such as by using geriatric assessment tools, frailty practitioners were able to avoid hospital admission for some PLWD, including by linking patients to community-based resources (Q7 and Q8 in Table 2), and reduce length of stay (Q9).

#### Integrated Dementia Care

Clinician respondents described the benefits of integrating different institutional resources to meet PLWD’s complex needs. Examples included a team providing short-term hospital-level treatments at home (Q10 in Table 2) and specialized care homes (equivalent to US nursing homes) modeled after the Enhanced Health in Care Homes framework (Q11 and Q12).^31,32^ By bridging inpatient and primary care services, these services could shorten length of stay (Q11) and reduce admissions (Q12).

### System-Level Factors

System-level factors encompassed social, economic, political, and legal influences on the health care system. This included national health policies, legislation, regulatory frameworks, and cultural norms. Both cohorts spoke to factors at this level.

#### Empowered Paramedics

Clinician respondents described how paramedics were empowered to act on documented advance care plans and engage in goals-of-care discussions (Q13 and Q14 in Table 2). Caregiver respondents further recounted how paramedics guided in-the-moment decision-making to deescalate care in the community (Q15 and Q16). Paramedics provided alternative options when hospitalization appeared goal discordant and implemented families’ preferences (Q15 and Q16).

#### Shared Electronic Record Systems

Clinician respondents referenced local versions of the nationalized Electronic Palliative Care Coordinating Systems (EPaCCS), which enabled electronic information sharing across clinicians within a geographical region, including urgent care plans and do-not-resuscitate orders. Clinician respondents reported that EPaCCS could prevent goal-discordant escalation (Q17 in Table 2). In the community, communicating urgent care plans through EPaCCS was reported to help care home staff understand a patient might be nearing the end of life. Depending on the recorded care plan, EPaCCS access could avoid hospitalization (Q18).

#### Treatment Escalation Plans

Clinician respondents emphasized establishing treatment “ceilings” for PLWD based on clinical status and patient preferences using treatment escalation plans (TEPs) (Q19 in Table 2). TEPs documented personalized escalation recommendations and were issued for all patients at the trust. TEPs guided clinician-led consensus decisions at critical moments (eg, transitions), helping to identify when a patient’s condition was unlikely to be reversible and set escalation parameters accordingly (Q20).

#### Legal and Policy Frameworks

Clinician respondents reflected that UK laws (eg, Mental Capacity Act 2005) and clinical practice guidelines (eg, General Medical Council’s Decision-Making and Consent) supported clinician-led best interest decision-making when patients lack decisional capacity.^33,34^ While clinician respondents stipulated that patient preferences were essential to consider, responsibility for how treatment ought to proceed was reported to rest with the most senior clinician and multidisciplinary team consensus, reflective of UK laws and practice guidelines (Q21, Q22, and Q23 in Table 2).^33,34^ Clinician respondents reported that families were entitled to second opinions but could not override decisions without legal action (Q23 and Q24).

While caregivers did not refer directly to laws or guidelines, their perspectives reflected the legal and policy context clinicians described. Some caregiver respondents endorsed clinicians’ expertise in decision-making (Q25), while others noted difficulties with a clinician-led model. These included feeling unheard (Q26), failure to meet patients’ holistic needs (Q27), and concerns that the system might not account for different cultural values (Q28 and Q29).

#### Treatment Intensity Norms for PLWD

Clinician respondents described a norm toward lower-intensity treatment for PLWD, notably in preventing escalation when they judged that expected harms outweighed benefits. Escalation from care homes to hospital (Q30 in Table 2) was reported to be unusual. Furthermore, PLWD with baseline frailty and minimally or nonreversible pathology were reported to rarely be ICU candidates (Q31 and Q32). Nevertheless, caregiver respondents worried that pursuing lower-intensity treatments could lead clinicians to overlook reversible decompensation and prematurely withhold treatments (Q33 and Q34).

#### Resource Allocation

Clinician respondents emphasized anticipatory care as a preventative measure to avoid unnecessary hospitalizations among PLWD. This approach was partly framed as optimizing resource utilization within the NHS’s constraints (Q35 in Table 2). Yet, some caregiver respondents noted that limited resources could interfere with desired limits on escalation. For example, one patient was unable to access palliative care due to restricted NHS trust resources, leading to a potentially avoidable hospitalization near the end of life (Q36).

## Discussion

We identified factors at the individual, institutional, and system levels of the British health care system that appeared to interact to generate a clinical practice pattern that oriented away from potentially nonbeneficial treatment escalation for PLWD. Studies suggest that increasing treatment intensity toward the end of life is the default trajectory in the United States. Treatments are often administered, particularly in the ICU, “without pause or design.”^35^ This pattern, conceptualized as clinical momentum, often leads US clinicians, patients, and their families to tolerate escalating treatment without due consideration of likely outcomes or goal concordance.^11^ US studies have shown that even when clinicians recognize high-intensity LSTs as potentially nonbeneficial, they have limited ability to resist clinical momentum if factors at the institutional and system levels do not support them.^8,9,10^

The global culture of biomedicine often encourages clinicians to do something, an influence to which Great Britain is not immune.^36^ Nevertheless, our data suggest that the observed factors in the British health care system worked together to prevent a default tendency toward treatment escalation in PLWD, especially when it stood to be nonbeneficial or goal-discordant. We term this phenomenon, which contrasts with clinical momentum, *clinical deceleration* (Figure).

**Figure.**
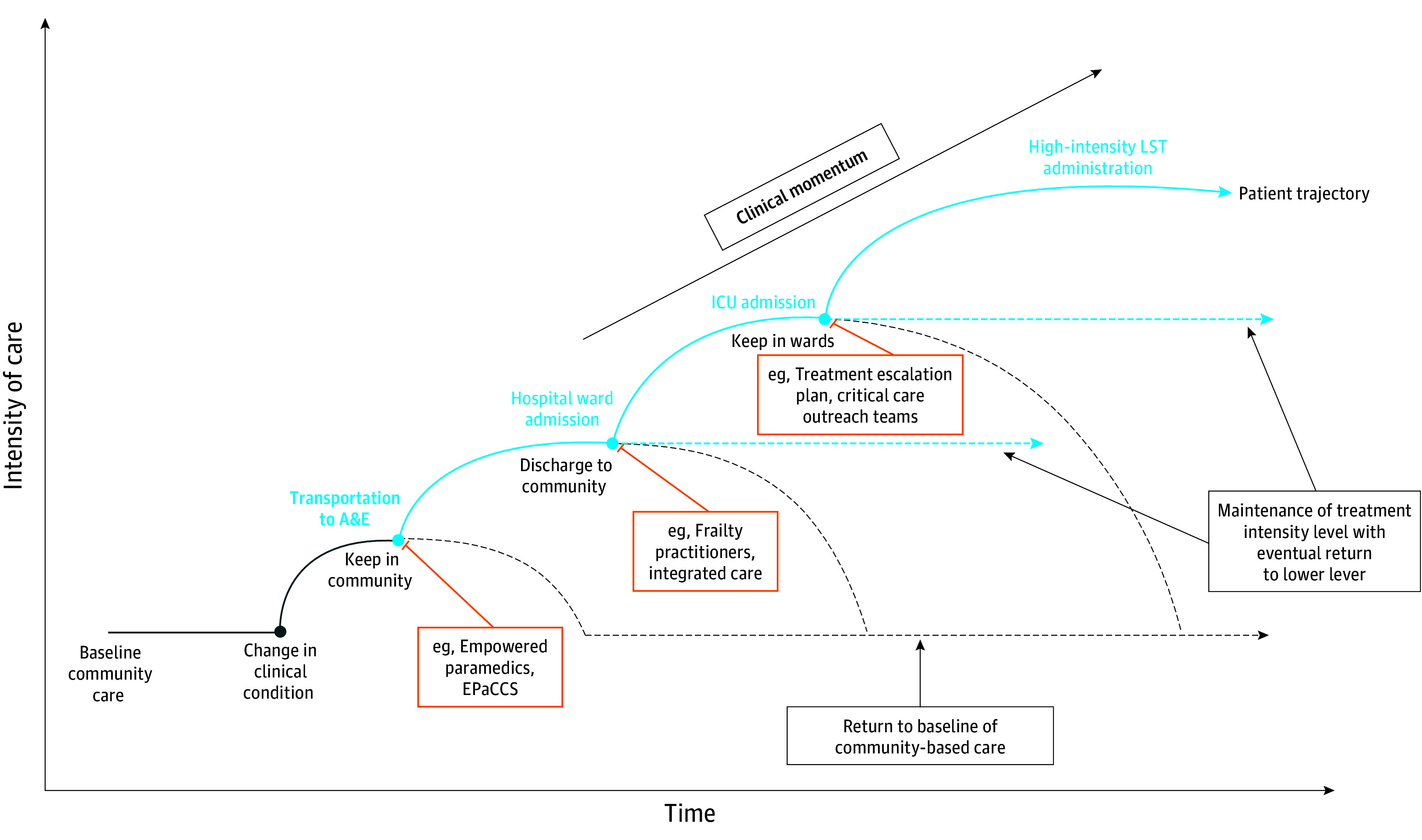
Hypothetical Examples of Patient Trajectories for People Living With Advanced Dementia Decision-making nodes (represented by blue dots) are punctuated throughout the care trajectories. The curves following each node are examples of different patient trajectories: treatment escalation (solid blue curves), maintenance of the same levels of treatment intensity (dotted light blue lines), or decreases and eventual returns to the treatment intensity observed at baseline community care (dotted gray curves). A&E indicates accident and emergency; EPaCCS, Electronic Palliative Care Coordinating Systems; ICU, intensive care unit; and LST, life-sustaining treatment.

We hypothesize that clinical deceleration reflected factors working together to provide structured opportunities for intentional (de-)escalation decisions. System-level factors appeared to shape institutional arrangements, such as through the operationalization of system-level policies, like EPaCCS^37,38,39^ and TEPs,^40,41^ which may promote adherence to patient preferences and reduce unwanted treatments.^41,42,43,44^ Clinician respondents connected system-level policies to health care system objectives centered on anticipatory care delivery, including strengthening community-based care, reducing unnecessary hospitalizations, and encouraging early escalation discussions, all goals outlined by the NHS Long-Term Plan.^45^ When implemented across institutional settings (eg, community, A&E, and inpatient wards), complementary system-level policies created opportunities before and during hospitalizations for thoughtful pauses to assess ongoing treatment or escalation and proceed intentionally.^46^

Moreover, system-level laws, guidelines, and cultural norms were reported to shape the expectations of clinicians and caregivers in ways facilitating clinical deceleration. These interactions between the system and individual levels appeared to occur directly as well as mediated through the institutional level. Clinician respondents noted that national laws and guidelines placed decision-making responsibility for people lacking decisional capacity on clinicians; caregiver respondents agreed that clinicians should hold a central role.^33,34^ Both clinician and caregiver respondents described societal expectations and accepted clinical practices consistent with the view that ICU admission may have limited utility and ethical permissibility for PLWD, given unclear benefits and attendant harms.^47^ This appeared to align with system-level guidance in Great Britain.^34,48^

In the eTable in Supplement 1, we ground each of our major findings within the literature. While each factor looked to prevent escalating treatments when potentially nonbeneficial or goal-discordant independently, their cumulative effects collectively accounted for clinical deceleration in PLWD. Our results suggest that interactions between factors at different levels create a recursive feedback loop in which factors reproduce and strengthen each other over time.^10,49^ The eFigure in Supplement 1 presents our conceptual representation of these recursive interactions. Existing frameworks regarding decision-making in serious illness care have utilized similar socio-ecological approaches to conceptualize how factors at different health care system levels promote treatment escalation.^50,51,52^ To our knowledge, ours is the first empirically grounded framework that draws on this approach to conceptualize how multilevel factors may prevent potentially nonbeneficial escalation.

While multilevel factors appeared to prevent a default tendency toward escalation, they held drawbacks. First, caregiver respondents worried that system-level factors like clinician-led decision-making, cultural norms, and resource allocation could lead to missed opportunities for benefit. Indeed, many of the factors described could lead to undertreatment of PLWD.^53,54,55^ This risk underscores the need to ensure that system-level priorities do not discourage beneficial treatments nor override individualized decision-making.^56,57^ Second, data evaluating the effectiveness of system- and institutional-level factors in reducing escalation remain limited due to variable implementation and evaluation across Great Britain. For example, EPaCCS,^37,39,58^ acute frailty services^59,60^—including frailty practitioners—and ICU outreach teams^48^ all vary in adoption. Nonetheless, some studies suggest benefits, with acute frailty services reducing admissions^59^ and TEPs associated with fewer potentially nonbeneficial treatments.^41^

### Limitations

This study has several limitations. First, clinician respondents were recruited from a single NHS trust in South London, so their perspectives on institutional-level factors and the implementation of system-level policies may not be representative of conditions in other NHS trusts or regions. Thus, clinical deceleration may manifest differently in other regions, if at all. Second, caregiver respondents were drawn primarily through 2 organizations focused on dementia and palliative care. Their involvement in these networks may have shaped their knowledge and perspectives. Additionally, our elucidation of system-level factors shaping escalation does not reflect the perspectives of health care system leaders. Although our approach enabled us to construct one view of how system-level factors operate, we recognize this is partial and may miss relevant insights into system function.

## Conclusions

In this qualitative study of clinicians and caregivers of PLWD in Great Britian, respondents reported individual-, institutional-, and system-level factors that, in aggregate, appeared to prevent potentially nonbeneficial treatment escalation and to facilitate deescalation when preferred, a phenomenon we termed clinical deceleration. Future research could engage health care system leaders, explore whether a similar clinical deceleration pattern extends to other regions in Great Britain or among patients with conditions other than dementia, and interrogate the influence of other system-level factors, such as the increasing role of financial actors in health care, on end-of-life treatment intensity.^61,62^ Comparative analyses could explore differences between high- and low-intensity systems, such as the United States and Great Britain, in greater depth. Understanding mechanisms promoting clinical deceleration will be vital for clarifying differences between health care systems and developing interventions to prevent default escalation toward potentially nonbeneficial treatments in the United States and other high-intensity systems.

## Notes

Supplement 1. **eTable.** Qualitative Findings Compared With Existing Literature
eReferences.
**eFigure.** Conceptual Representation of Study Findings Illustrating How Multilevel Factors Interact to Produce Clinical Deceleration

Supplement 2.
Data Sharing Statement
