# Supplementary material for: Systemic Strategies to Prevent Nonbeneficial Treatments Near the End of Life
Source: JAMA Netw Open. 2025 Jul 10;8(7):e2519771. doi: 10.1001/jamanetworkopen.2025.19771 (PMC12246877; doi:10.1001/jamanetworkopen.2025.19771)
Supplement: Supplement 1. — eTable. Qualitative Findings Compared With Existing Literature eReferences. eFigure. Conceptual Representation of Study Findings Illustrating How Multilevel Factors Interact to Produce Clinical Deceleration [file jamanetwopen-e2519771-s001.pdf]

Supplemental Online Content

Weiss Goitiandia S, Sun AZ, Rosenwohl-Mack A, et al. Systemic strategies to prevent nonbeneficial treatments near the end of life. *JAMA Netw Open.* 2025;8(7):e2519771. doi:10.1001/jamanetworkopen.2025.19771

eTable. Qualitative Findings Compared With Existing Literature

eReferences.

eFigure. Conceptual Representation of Study Findings Illustrating How Multilevel Factors Interact to Produce Clinical Deceleration

This supplemental material has been provided by the authors to give readers additional information about their work.

© 2025 Weiss Goitiandia S et al. *JAMA Network Open*.

eTable. Qualitative Findings Compared With Existing Literature

Main Finding	Relevant Literature	Great Britain-Specific Adaptation	References
Clinicians' transparent communication facilitated careful decisions to escalate or de-escalate	In a systematic review of qualitative data mostly from the US, Anderson et al.[1] found that transparent communication with caregivers (e.g., acknowledging deterioration, tailoring information, and highlighting adverse outcomes) in the ICU facilitated decisions to transition patients approaching the end of life to comfort care.	Our findings appear consistent with prior literature on caregiver-clinician communication, which suggest that clear and honest communication may constructively support decisions not to escalate or to de-escalate goal-discordant or non-beneficial treatments.	1. Anderson RJ, Bloch S, Armstrong M, Stone PC, Low JT. Communication between healthcare professionals and relatives of patients approaching the end-of-life: A systematic review of qualitative evidence. Palliat Med. 2019;33(8):926-941. doi:10.1177/0269216319852007
Understanding the dementia trajectory empowered caregivers to de-escalate treatments	Cajavilca and Sadarangani[2] reviewed studies on dementia literacy among caregivers of PLWD in the US. This study considered caregivers 'literate' if they had the knowledge needed for informed decision-making, as well as to identify care gaps and access resources for long-term care. Dementia literacy among the caregivers of PLWD led to fewer avoidable hospitalizations. Recognition by caregivers of dementia as a terminal illness led to increased utilization of aging-in-place resources and hospice care.	The studies reviewed align with our findings and further demonstrate that caregiver knowledge of the dementia disease trajectory (e.g., recognition of dementia as a terminal disease and awareness of likely outcomes after hospitalization) may be an important factor in limiting escalations of treatment that may be non-beneficial or unwanted.	2. Cajavilca M, Sadarangani T. Dementia-literate informal caregivers: An evolutionary concept analysis. Nursing Outlook. 2024;72(5):102224. doi:10.1016/j.outlook.2024.102224
Pre-ICU pathways enabled clinician-led, consensus-driven decisions on ICU admissions	Previous ethnography work by Pattison et al.[3] exploring decision-making in serious illness care in GB found that Critical Care Outreach Teams (CCOTs, equivalent to US Rapid Response Teams (RRTs)) played a crucial role in initiating goals-of-care conversations and facilitating escalation decisions early in hospitalization, including to prevent escalation when potentially non-beneficial. In the US, although RRTs have been presented as a strategy to avoid unwanted ICU admissions,[4] evidence of their effectiveness is mixed: they have been found to have no significant effect on the proportion of patients who had family conferences or orders to limit LST, and patients who interfaced with teams were less likely to receive palliative care	Consistent with Pattison et al.,[3] our results demonstrate that CCOTs (referred to as ICU outreach teams) may prevent potentially non-beneficial treatment escalation for PLWD by supporting early conversations about escalation and providing an opportunity for the clinical team to achieve consensus. Clinicians reported using this service to determine for which clinical settings (e.g., high dependency units, ICU) individual PLWD might be candidates.	3. Pattison N, Mclellan J, Roskelly L, McLeod K, Wiseman T. Managing clinical uncertainty: An ethnographic study of the impact of critical care outreach on end-of-life transitions in ward-based critically ill patients with a life-limiting illness. Journal of Clinical Nursing. 2018;27(21-22):3900-3912. doi:10.1111/jocn.14618<br><br>4. Khandelwal N, Long AC, Lee RY, McDermott CL, Engelberg RA, Curtis JR. Pragmatic methods to avoid intensive care unit admission when it does not align with patient and family goals. The Lancet Respiratory Medicine. 2019;7(7):613-625. doi:10.1016/S2213-2600(19)30170-5<br><br>5. Downar J, Barua R, Rodin D, et al. Changes in end of life care 5 years after the introduction of a rapid response team: A multicentre retrospective study. Resuscitation. 2013;84(10):1339-1344.

© 2025 Weiss Goitiandia S et al. *JAMA Network Open*.

	consultations.[5] One US study demonstrated that early identification of patients as at high risk for deterioration (i.e., likely to need consultation) did lead to decreases in unplanned and unnecessary ICU admissions for people living with serious illness.[6]		doi:10.1016/j.resuscitation.2013.03.003<br><br>6. Danesh V, Neff D, Jones TL, et al. Can proactive rapid response team rounding improve surveillance and reduce unplanned escalations in care? A controlled before and after study. International Journal of Nursing Studies. 2019;91:128-133. doi:10.1016/j.ijnurstu.2019.01.004
Frailty practitioners as an institutional decision-making resource	Prior studies in Ireland[7] and the US[8] show that standardized use of comprehensive geriatric assessments conducted by dedicated geriatrician-led multidisciplinary teams can reduce ED stays, lower hospital readmissions, and improve quality of life. Further evidence suggests that specialist-led ED models may help streamline triage, leading to reductions in hospital admissions[9] and decreasing length of hospital stays.[10] However, these studies do not address specific roles like those of the frailty practitioner in our study. Further, some US studies highlight inconsistencies in the use of comprehensive geriatric assessments (especially by non-specialized users), with nursing assessments showing no significant impact on hospitalizations or readmissions and, in some cases, increasing ED visits.[11]	Although prior studies demonstrate how the standardized usage of a comprehensive geriatric assessment may help prevent escalation, the literature does not examine frailty practitioners' (or an equivalent) unique role, which includes collating documents such as advance care plans and 'Do-Not-Attempt-Resuscitation' orders, conducting geriatric assessments in A&E, and coordinating transitions to community care. Our respondents emphasized these responsibilities as important factors supporting the prevention of unnecessary hospital admissions. Further, while US-based work by Jennings et al.[10] examines nurse practitioner-led care coordination for PLWD, their work focuses on outpatient nurses. In contrast, frailty practitioners are deployed specifically in the A&E and implemented at the institutional level, unlike more siloed models of coordinated care.	7. Leahy A, Barry L, Corey G, et al. Frailty screening with comprehensive geriatrician-led multidisciplinary assessment for older adults during emergency hospital attendance in Ireland (SOLAR): a randomised controlled trial. The Lancet Healthy Longevity. 2024;5(11):100642. doi:10.1016/j.lanhl.2024.100642<br><br>8. Grudzen C, Richardson LD, Baumlin KM, et al. Redesigned geriatric emergency care may have helped reduce admissions of older adults to intensive care Units. Health Aff (Millwood). 2015;34(5):788-795. doi:10.1377/hlthaff.2014.0790<br><br>9. Wang DH, Heidt R. Emergency department embedded palliative care service creates value for health systems. J Palliat Med. 2023;26(5):646-652. doi:10.1089/jpm.2022.0245<br><br>10. Jennings LA, Turner M, Keebler C, et al. The effect of a comprehensive dementia care management program on end-of-life care. J Am Geriatr Soc. 2019;67(3):443-448. doi:10.1111/jgs.15769<br><br>11. Malik M, Moore Z, Patton D, O'Connor T, Nugent LE. The impact of geriatric focused nurse assessment and intervention in the emergency department: A systematic review. International Emergency Nursing. 2018;37:52-60. doi:10.1016/j.ienj.2018.01.008
Integrated dementia care across the	In the US, existing comprehensive dementia care programs seek to develop individual care plans, monitor and adapt	Our findings align with US literature indicating that integrated and timely dementia care, supported by community	12. Tan ZS, Qureshi N, Spivack E, et al. Pragmatic implementation of comprehensive dementia care management: The Cedars-Sinai C.A.R.E.S. Program

© 2025 Weiss Goitiandia S et al. *JAMA Network Open*.

institution prevented unnecessary hospitalizations	care plans, make referrals to community services, and offer 24/7 access to support lines as well as to provide telehealth appointments and connections to community-based clinicians. These services have been shown to improve care coordination for PLWD, decreasing ED visits and hospital admissions and increasing advance care planning uptake and hospice enrollment.[12,13] Another study found that similar coordinated care programs designed for nursing home residents, in addition to increases in nursing staff, led to fewer hospitalizations among residents.[14]	clinicians (e.g., specialized care home GPs) may help prevent unnecessary hospitalizations for PLWD and enable patients to return more quickly to the community if hospitalized. While coordinated care programs exist in the US, they often do not include as many services as reported in our data: clinicians reported that '@home' care teams could provide PLWD with short-term hospital-level interventions (e.g., IV antibiotics, oxygen admission) at home. Comprehensive dementia care programs in the US are typically implemented at the institutional level. Systemic programs (e.g., Medicare programs) designed for older adults, including PLWD, cover limited home health services and often do not cover coordinated care services.[13]	preliminary data. Journal of the American Geriatrics Society. 2024;72(8):2532-2543. doi:10.1111/jgs.18891<br><br>13. Samus QM, Davis K, Willink A, et al. Comprehensive home-based care coordination for vulnerable elders with dementia: Maximizing Independence at Home-Plus—Study protocol. Int J Care Coord. 2017;20(4):123-134. doi:10.1177/2053434517744071<br><br>14. Ingber MJ, Feng Z, Khatutsky G, et al. Initiative to reduce avoidable hospitalizations among nursing facility residents shows promising results. Health Aff (Millwood). 2017;36(3):441-450. doi:10.1377/hlthaff.2016.1310
Paramedics were empowered in their role to implement decisions not to escalate treatments	Studies have shown that paramedics are more confident in decisions not to escalate treatments when they have access to clear patient preferences, supportive policies, and structured decision-making frameworks.[15] Electronic registries and policy changes allowing paramedics to halt resuscitation have further helped match treatment delivered with patient wishes.[16] However, challenges include limited access to up-to-date patient information, external pressure from nursing staff and families, and variability in paramedic decision-making.[17]	Our findings align with existing studies, reinforcing that supportive policies and accessible electronic advance care plans can help paramedics provide goal-concordant care and reduce potentially non-beneficial escalations of treatment. In particular, paramedics, when empowered by policy to use documented care plans to make escalation decisions in the field, may prevent hospitalization when this appears misaligned with a patient's goals or best interests. Notably, while US-based studies have similar findings, these results were only observed in very few regions – such as Los Angeles and Oregon – boasting novel policies specifically designed to empower paramedics.	15. Schmidt TA, Olszewski EA, Zive D, Fromme EK, Tolle SW. The Oregon Physician orders for life-sustaining treatment registry: A preliminary study of emergency medical services utilization. J Emerg Med. 2013;44(4):796-805. doi:10.1016/j.jemermed.2012.07.081<br><br>16. Grudzen CR, Timmermans S, Koenig WJ, et al. Paramedic and emergency medical technicians views on opportunities and challenges when forgoing and halting resuscitation in the field. Acad Emerg Med. 2009;16(6):532-538. doi:10.1111/j.1553-2712.2009.00427.x<br><br>17. Murphy-Jones G, Timmons S. Paramedics' experiences of end-of-life care decision making with regard to nursing home residents: an exploration of influential issues and factors. Emerg Med J. 2016;33(10):722-726. doi:10.1136/emermed-2015-205405
Shared electronic record systems conveying	EPaCCS is a UK-wide initiative created in response to the UK Government's 2008 'End-of-Life Care Strategy,'[18] aiming to	As observed in Oregon, our results suggest that sharing health records among emergency personnel, including	18. Department of Health. End of Life Care Strategy: Promoting High Quality Care for All Adults at the End of Life.; 2008.

© 2025 Weiss Goitiandia S et al. *JAMA Network Open*.

patients' preferences aided goal-concordant care	document and share patients' care preferences across care settings. Despite successes, such as enabling most patients to die in their preferred location,[19] EPaCCS has faced implementation challenges like inconsistent use across settings, technical limitations, and a lack of trust in record accuracy. While clinicians recognized EPaCCS' potential, sustained engagement and partial redesign were identified as future directions to improve effectiveness.[19,20]<br><br>Additionally, we found data about a similar electronic system in the US, the Oregon POLST registry. Studies on its use have shown that access improved EMS adherence to patient preferences and reduced unwanted interventions.[15,21]	paramedics, may promote goal-concordant care for PLWD and prevent escalation if avoiding hospitalization aligns with expressed preferences. However, compared to the Oregon POLST, which only documents select treatment preferences during a medical emergency,[15] EPaCCS records provided clinicians with more granular details, such as diagnoses, preferred place of death, goals of care, and 'ceilings of care,' established by other clinicians who had previously interfaced with a patient. By sharing this information, EPACCS records could support paramedics' adherence to previously established plans and prevent unwanted or non-beneficial treatment escalation. Although clinician respondents viewed EPaCCS as an effective tool in the care of PLWD, the existing literature suggests its success is contingent upon how well it is implemented.	19. Petrova M, Riley J, Abel J, Barclay S. Crash course in EPaCCS (Electronic Palliative Care Coordination Systems): 8 years of successes and failures in patient data sharing to learn from. BMJ Support Palliat Care. 2018;8(4):447-455. doi:10.1136/bmjspcare-2015-001059<br><br>20. Bradshaw A, Birtwistle J, Evans CJ, et al. Factors influencing the implementation of digital advance care planning: Qualitative Interview Study. J Med Internet Res. 2024;26(1):e50217. doi:10.2196/50217<br><br>15. Schmidt TA, Olszewski EA, Zive D, Fromme EK, Tolle SW. The Oregon Physician orders for life-sustaining treatment registry: A preliminary study of emergency medical services utilization. J Emerg Med. 2013;44(4):796-805. doi:10.1016/j.jemermed.2012.07.081<br><br>21. Richardson DK, Fromme E, Zive D, Fu R, Newgard CD. Concordance of out-of-hospital and emergency department cardiac arrest resuscitation with documented end-of-life choices in Oregon. Ann Emerg Med. 2014;63(4):375-383. doi:10.1016/j.annemergmed.2013.09.004
Treatment Escalation Plans (TEPs) prevented escalation to potentially non-beneficial LST	Introduced to the National Health Service (NHS) in 2006, TEPs have been shown to reduce non-beneficial ICU admission, LST administration, and associated harms by outlining clinically appropriate levels of care and ensuring early decision-making.[22–25] Studies have demonstrated that TEPs improve communication among healthcare teams, increase multidisciplinary engagement, and enhance clarity in treatment goals[23,26] However, some studies caution that TEPs are primarily clinician-driven; Warner et al.[22] highlighted that while clinicians aimed for optimal medical outcomes, patient perspectives were rarely prioritized.	With no direct equivalents in the US to our knowledge, TEPs are a novel factor in GB that may help prevent treatment escalation, especially to potentially non-beneficial high-intensity LST. Clinicians reported that TEPs documented 'ceilings of care' and were consulted at critical moments in the care of PLWD, which prevented non-beneficial escalation and improved care coordination, aligning with the literature.	22. Warner BE, Wells M, Vindrola-Padros C, Brett SJ. Shared decision-making with older people on TReatment Escalation planning for Acute deterioration in the emergency Medical Setting: a qualitative study of Clinicians' perspectives (STREAMS-C). Age Ageing. 2024;53(9):afae204. doi:10.1093/ageing/afae204<br><br>23. Taylor DR, Lightbody CJ, Venn R, Ireland AJ. Responding to the deteriorating patient: The rationale for treatment escalation plans. Journal of the Royal College of Physicians of Edinburgh. 2022;52(2):172-179. doi:10.1177/14782715221103390<br><br>24. Fadel MG, Parekh K, Hayden P, Krishnan P. Improving resuscitation decisions: a trust-wide

© 2025 Weiss Goitiandia S et al. *JAMA Network Open*.

			initiative. BMJ Open Qual. 2018;7(4):e000268. doi:10.1136/bmjoq-2017-000268<br><br>25. Lightbody CJ, Campbell JN, Herbison GP, Osborne HK, Radley A, Taylor DR. Impact of a treatment escalation/limitation plan on non-beneficial interventions and harms in patients during their last admission before in-hospital death, using the Structured Judgment Review Method. BMJ Open. 2018;8(10):e024264. doi:10.1136/bmjopen-2018-024264<br><br>26. Johnson M, Whyte M, Loveridge R, Yorke R, Naleem S. A unified electronic tool for CPR and emergency treatment escalation plans improves communication and early collaborative decision making for acute hospital admissions. BMJ Qual Improv Rep. 2017;6(1):u213254.w6626. doi:10.1136/bmjquality.u213254.w6626
Legal and policy frameworks supported clinician-led best-interest decision-making	The General Medical Council (GMC), an independent regulator of physicians in the UK, and the Mental Capacity Act law of 2005 (MCA) provide clinicians with guidance on decision-making.[27] The GMC's 'Decision Making and Consent' encourages clinicians to honor patient preferences by considering patients' values and beliefs and consulting care plans, family, carers, and advocates.[28] However, this guidance also contains language that urges clinicians not to provide a treatment if it does 'not serve the patient's needs' – even upon request. Further, the MCA is a legal framework that provides guidance for clinicians in circumstances where patients do not have the capacity to make decisions. Crucial to the MCA is language about clinicians making decisions, including regarding LST, that are in the 'best interest' of their patients rather than decisions motivated by preventing death.	Clinician respondents identified GMC guidance and the MCA as system-level policies and laws guiding their practice, including regarding escalation for PLWD. While espousing respect for patient preferences, respondents highlighted that caregivers did not have the right to demand treatments and emphasized that clinicians had legal backing in decisions to limit escalation if it was judged not to be in the patient's best interest. These UK-specific frameworks, which do not have a direct nationwide equivalent in the US, may suggest that legal and health system-level commitments to best-interest decision-making, as enshrined in policy frameworks and laws, may promote decisions to limit escalation for PLWD if treatments stand to be non-beneficial.	27. *Mental Capacity Act 2005*, c. 4. Available at: https://www.legislation.gov.uk/ukpga/2005/9/contents (Accessed 28 February 2025).<br><br>28. General Medical Council. Decision Making and Consent. General Medical Council; 2020. https://www.gmc-uk.org/professional-standards/the-professional-standards/decision-making-and-consent (Accessed February 28, 2025).

© 2025 Weiss Goitiandia S et al. *JAMA Network Open*.

A tendency toward lower treatment intensity for PLWD	Survey work investigating the admission preferences of UK-based intensivists found that a patient's age was the most significant factor impacting decisions regarding ICU admissions across the NHS.[29] This work, combined with other quantitative work demonstrating that older adults living with serious illness in the UK had lower rates of ICU admission[30] and lower rates of mechanical ventilation utilization[31] than in the US, suggests that clinicians in the UK may abide by norms (e.g., ICU admission criteria) that may tend toward less treatment escalation in older patients than in the US, including PLWD.	In our results, clinician respondents reified that PLWD are often viewed as a population in whom treatment escalation, particularly to the ICU, ought to be carefully considered, appealing to reasons such as low potential benefit due to disease severity and irreversibility alongside comorbidities and frailty. Thus, our results align with the existing literature and suggest that ICU admission in GB may be more dependent than in the US on whether patients, including PLWD, are thought by clinicians to be likely to benefit from admission and LST.	29. Bassford CR, Krucien N, Ryan M, et al. U.K. Intensivists' preferences for patient admission to ICU: evidence from a choice experiment. Crit Care Med. 2019;47(11):1522-1530. doi:10.1097/CCM.0000000000003903<br><br>30. Wunsch H, Angus DC, Harrison DA, Linde-Zwirble WT, Rowan KM. Comparison of medical admissions to intensive care units in the United States and United Kingdom. Am J Respir Crit Care Med. 2011;183(12):1666-1673. doi:10.1164/rccm.201012-1961OC<br><br>31. Jivraj NK, Hill AD, Shieh MS, et al. Use of Mechanical Ventilation Across 3 Countries. JAMA Intern Med. 2023;183(8):824. doi:10.1001/jamainternmed.2023.2371
Concerns about resource allocation influenced approaches to treatment escalation	In a scoping review of quantitative papers across the US, Delamater et al.[32] found that an increase in hospital beds was associated with higher rates of hospital admissions. Regarding PLWD specifically, Teno et al.[33] found that increases in the availability of ICU beds were associated with increases in ICU admissions for PLWD. In conjunction, these findings suggest that increased availability of resources may promote more frequent treatment escalation in PLWD, at least in the US.	Respondents reported that the prudent allocation of limited resources in settings such as the NHS, a publicly funded nationalized healthcare system, encouraged anticipatory planning. This planning sought to prevent patients, including PLWD, from reaching crisis points requiring in-extremis decision-making, which may have supported preventing treatment escalation as a default. Thus, our findings suggest that just as increased availability of resources (e.g., ICU beds) may be related to increased treatment intensity, limited availability of resources may encourage judicious treatment escalation decisions. Yet, limited resources may also lead to the undertreatment of patients in GB.	32. Delamater PL, Messina JP, Grady SC, WinklerPrins V, Shortridge AM. Do more hospital beds lead to higher hospitalization rates? A spatial examination of Roemer's Law. PLoS ONE. 2013;8(2):e54900. doi:10.1371/journal.pone.0054900<br><br>33. Teno JM, Gozalo P, Khandelwal N, et al. Association of increasing use of mechanical ventilation among nursing home residents with advanced dementia and intensive care unit beds. JAMA Intern Med. 2016;176(12):1809. doi:10.1001/jamainternmed.2016.5964

© 2025 Weiss Goitiandia S et al. *JAMA Network Open*.

eReferences.

1. Anderson RJ, Bloch S, Armstrong M, Stone PC, Low JT. Communication between healthcare professionals and relatives of patients approaching the end-of-life: A systematic review of qualitative evidence. Palliat Med. 2019;33(8):926-941. doi:10.1177/0269216319852007

2. Fernandez Cajavilca M, Sadarangani T. Dementia-literate informal caregivers: An evolutionary concept analysis. Nurs Outlook. 2024;72(5):102224. doi:10.1016/j.outlook.2024.102224

3. Pattison N, Mclellan J, Roskelly L, McLeod K, Wiseman T. Managing clinical uncertainty: An ethnographic study of the impact of critical care outreach on end-of-life transitions in ward-based critically ill patients with a life-limiting illness. J Clin Nurs. 2018;27(21-22):3900-3912. doi:10.1111/jocn.14618

4. Khandelwal N, Long AC, Lee RY, McDermott CL, Engelberg RA, Curtis JR. Pragmatic methods to avoid intensive care unit admission when it does not align with patient and family goals. Lancet Respir Med. 2019;7(7):613-625. doi:10.1016/S2213-2600(19)30170-5

5. Downar J, Barua R, Rodin D, et al. Changes in end of life care 5 years after the introduction of a rapid response team: A multicentre retrospective study. Resuscitation. 2013;84(10):1339-1344. doi:10.1016/j.resuscitation.2013.03.003

6. Danesh V, Neff D, Jones TL, et al. Can proactive rapid response team rounding improve surveillance and reduce unplanned escalations in care? A controlled before and after study. Int J Nurs Stud. 2019;91:128-133. doi:10.1016/j.ijnurstu.2019.01.004

7. Leahy A, Barry L, Corey G, et al. Frailty screening with comprehensive geriatrician-led multidisciplinary assessment for older adults during emergency hospital attendance in Ireland (SOLAR): a randomised controlled trial. Lancet Healthy Longev. 2024;5(11):100642. doi:10.1016/j.lanhl.2024.100642

8. Grudzen C, Richardson LD, Baumlin KM, et al. Redesigned geriatric emergency care may have helped reduce admissions of older adults to intensive care units. Health Aff (Millwood). 2015;34(5):788-795. doi:10.1377/hlthaff.2014.0790

9. Wang DH, Heidt R. Emergency department embedded palliative care service creates value for health systems. J Palliat Med. 2023;26(5):646- 652. doi:10.1089/jpm.2022.0245

10. Jennings LA, Turner M, Keebler C, et al. The fffect of a comprehensive dementia care management program on end-of-life care. J Am Geriatr Soc. 2019;67(3):443-448. doi:10.1111/jgs.15769

11. Malik M, Moore Z, Patton D, O'Connor T, Nugent LE. The impact of geriatric focused nurse assessment and intervention in the emergency department: A systematic review. Int Emerg Nurs. 2018;37:52-60. doi:10.1016/j.ienj.2018.01.008

12. Tan ZS, Qureshi N, Spivack E, et al. Pragmatic implementation of comprehensive

© 2025 Weiss Goitiandia S et al. *JAMA Network Open*.

dementia care management: The Cedars-Sinai C.A.R.E.S. Program preliminary data. J Am Geriatr Soc. 2024;72(8):2532-2543. doi:10.1111/jgs.18891

13. Samus QM, Davis K, Willink A, et al. Comprehensive home-based care coordination for vulnerable elders with dementia: Maximizing Independence at Home-Plus—Study protocol. Int J Care Coord. 2017;20(4):123-134. doi:10.1177/2053434517744071

14. Ingber MJ, Feng Z, Khatutsky G, et al. Initiative to reduce avoidable hospitalizations among nursing facility residents shows promising results. Health Aff (Millwood). 2017;36(3):441-450. doi:10.1377/hlthaff.2016.1310

15. Schmidt TA, Olszewski EA, Zive D, Fromme EK, Tolle SW. The Oregon physician orders for life-sustaining treatment registry: A preliminary study of emergency medical services utilization. J Emerg Med. 2013;44(4):796-805. doi:10.1016/j.jemermed.2012.07.081

16. Grudzen CR, Timmermans S, Koenig WJ, et al. Paramedic and emergency medical technicians views on opportunities and challenges when forgoing and halting resuscitation in the field. Acad Emerg Med. 2009;16(6):532-538. doi:10.1111/j.1553-2712.2009.00427.x

17. Murphy-Jones G, Timmons S. Paramedics' experiences of end-of-life care decision making with regard to nursing home residents: an exploration of influential issues and factors. Emerg Med J. 2016;33(10):722-726. doi:10.1136/emermed-2015-205405

18. Department of Health. End of Life Care Strategy: Promoting High Quality Care for All Adults at the End of Life.; 2008.

19. Petrova M, Riley J, Abel J, Barclay S. Crash course in EPaCCS (Electronic Palliative Care Coordination Systems): 8 years of successes and failures in patient data sharing to learn from. BMJ Support Palliat Care. 2018;8(4):447-455. doi:10.1136/bmjspcare-2015-001059

20. Bradshaw A, Birtwistle J, Evans CJ, et al. Factors influencing the implementation of digital advance care planning: qualitative interview Study. J Med Internet Res. 2024;26(1):e50217. doi:10.2196/50217

21. Richardson DK, Fromme E, Zive D, Fu R, Newgard CD. Concordance of out-of-hospital and emergency department cardiac arrest resuscitation with documented end-of-life choices in Oregon. Ann Emerg Med. 2014;63(4):375-383. doi:10.1016/j.annemergmed.2013.09.004

22. Warner BE, Wells M, Vindrola-Padros C, Brett SJ. Shared decision-making with older people on TReatment Escalation planning for Acute deterioration in the emergency Medical Setting: a qualitative study of Clinicians' perspectives (STREAMS-C). Age Ageing. 2024;53(9):afae204. doi:10.1093/ageing/afae204

23. Taylor DR, Lightbody CJ, Venn R, Ireland AJ. Responding to the deteriorating patient: The rationale for treatment escalation plans. J R Coll Physicians Edinb.

2022;52(2):172-179. doi:10.1177/14782715221103390

24. Fadel MG, Parekh K, Hayden P, Krishnan P. Improving resuscitation decisions: a trust-wide initiative. BMJ Open Qual. 2018;7(4):e000268. doi:10.1136/bmjoq-2017-000268

25. Lightbody CJ, Campbell JN, Herbison GP, Osborne HK, Radley A, Taylor DR. Impact of a treatment escalation/limitation plan on non-beneficial interventions and harms in patients during their last admission before in-hospital death, using the Structured Judgment Review Method. BMJ Open. 2018;8(10):e024264. doi:10.1136/bmjopen-2018-024264

26. Johnson M, Whyte M, Loveridge R, Yorke R, Naleem S. A unified electronic tool for CPR and emergency treatment escalation plans improves communication and early collaborative decision making for acute hospital admissions. BMJ Qual Improv Rep. 2017;6(1):u213254.w6626. doi:10.1136/bmjquality.u213254.w6626

27. Mental Capacity Act 2005, c. 4. Available at: https://www.legislation.gov.uk/ukpga/2005/9/contents (Accessed 28 February 2025).

28. General Medical Council. Decision Making and Consent. General Medical Council; 2020. https://www.gmc-uk.org/professional-standards/the- professional-standards/decision-making-and-consent (Accessed February 28, 2025).

29. Bassford CR, Krucien N, Ryan M, et al. U.K. Intensivists' preferences for patient admission to ICU: evidence from a choice experiment. Crit Care Med. 2019;47(11):1522-1530. doi:10.1097/CCM.0000000000003903

30. Wunsch H, Angus DC, Harrison DA, Linde-Zwirble WT, Rowan KM. Comparison of medical admissions to intensive care units in the United States and United Kingdom. Am J Respir Crit Care Med. 2011;183(12):1666-1673. doi:10.1164/rccm.201012-1961OC

31. Jivraj NK, Hill AD, Shieh MS, et al. Use of mechanical ventilation across 3 countries. JAMA Intern Med. 2023;183(8):824. doi:10.1001/jamainternmed.2023.2371

32. Delamater PL, Messina JP, Grady SC, WinklerPrins V, Shortridge AM. Do more hospital beds lead to higher hospitalization rates? A spatial examination of Roemer's Law. PLoS ONE. 2013;8(2):e54900. doi:10.1371/journal.pone.0054900

33. Teno JM, Gozalo P, Khandelwal N, et al. Association of increasing use of mechanical ventilation among nursing home residents With advanced Dementia and intensive care unit beds. JAMA Intern Med. 2016;176(12):1809. doi:10.1001/jamainternmed.2016.5964

© 2025 Weiss Goitiandia S et al. *JAMA Network Open*.

eFigure. Conceptual Representation of Study Findings Illustrating How Multilevel Factors Interact to Produce Clinical Deceleration

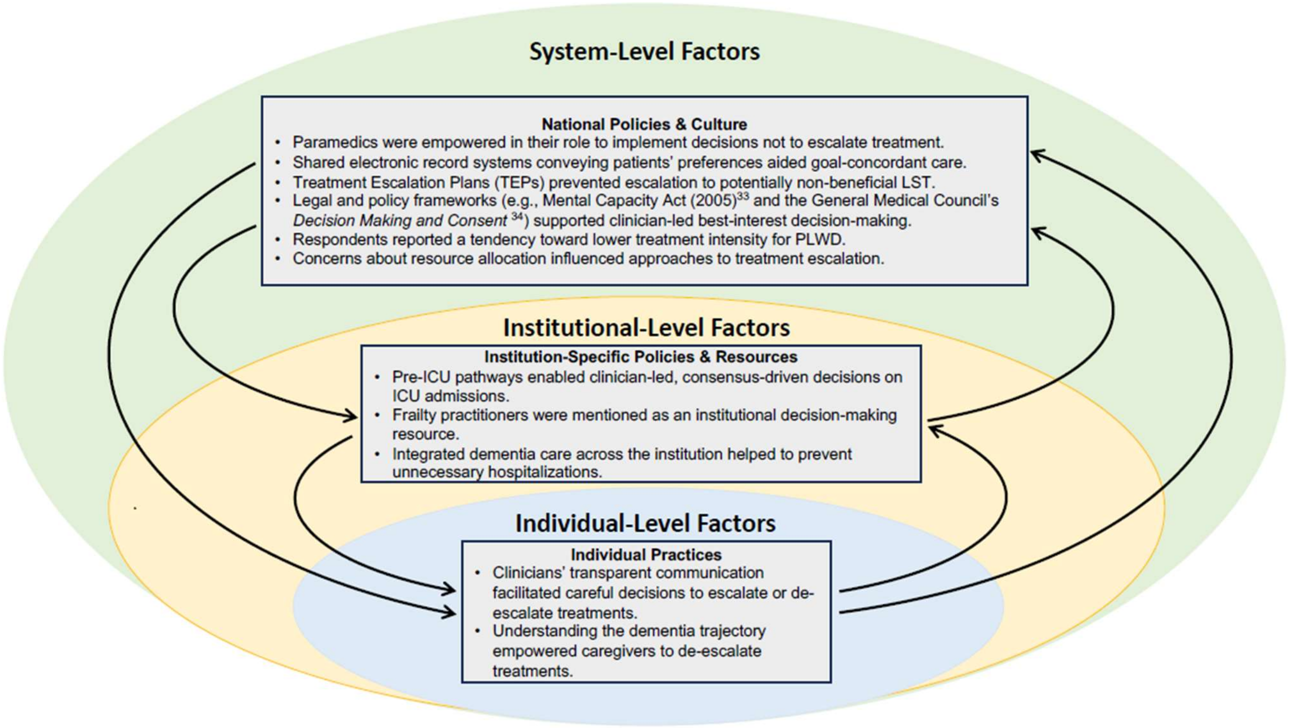

We posit that factors at different levels of Great Britain's healthcare system interacted and influenced each other (as represented by arrows) to contribute to clinical deceleration. For example, system-level factors (e.g., national policies and culture) influenced the development of institutional policies and allocation of institutional resources, which, in turn, affected how individual clinicians made decisions with PLWD and their caregivers. We hypothesize that factors at different levels influence each other in both directions through recursion, such that individual practices reflect and, in turn, reinforce institutional policies and practices and, eventually, national policies and culture.
