# Supplementary material for: Systemic Strategies to Prevent Nonbeneficial Treatments Near the End of Life
Source: JAMA Netw Open. 2025 Jul 10;8(7):e2519771. doi: 10.1001/jamanetworkopen.2025.19771 (PMC12246877; doi:10.1001/jamanetworkopen.2025.19771)
Supplement: Supplement 2. — Data Sharing Statement [file jamanetwopen-e2519771-s002.pdf]

Data Sharing Statement

Weiss Goitiandia. Systemic Strategies to Prevent Nonbeneficial Treatments Near the End of Life. *JAMA Netw Open*. Published July 10, 2025. doi:10.1001/jamanetworkopen.2025.19771

Data
Data available: No

Additional Information
Explanation for why data not available: This is a qualitative research project where the data are situated within the particular context of the project. Research participants have not consented to having their interview data shared beyond this particular study. Given the highly personal nature of qualitative research, it is impossible to fully anonymize the data, even if obviously identifying features (e.g., names) have been anonymized. If there are any potential collaborators interested in working on this study data, Dr Dzeng would need to add them to my IRB and/or obtain consent from the research participants. This would need to be on a case by case basis.
